# Brain-like hardware, do we need it?

**DOI:** 10.3389/fnins.2024.1465789

**Published:** 2024-12-16

**Authors:** Francesca Borghi, Thierry R. Nieus, Davide E. Galli, Paolo Milani

**Affiliations:** ^1^CIMAINA and Dipartimento di Fisica “A. Pontremoli”, Università degli Studi di Milano, Milan, Italy; ^2^Dipartimento di Scienze e Politiche Ambientali, Università degli Studi di Milano, Milan, Italy

**Keywords:** neuromorphic, unconventional computing, CMOS, nanoparticle networks, perceptron, hardware

## Abstract

The brain’s ability to perform efficient and fault-tolerant data processing is strongly related to its peculiar interconnected adaptive architecture, based on redundant neural circuits interacting at different scales. By emulating the brain’s processing and learning mechanisms, computing technologies strive to achieve higher levels of energy efficiency and computational performance. Although efforts to address neuromorphic solutions through hardware based on top-down CMOS-based technologies have obtained interesting results in terms of energetic efficiency improvement, the replication of brain’s self-assembled and redundant architectures is not considered in the roadmaps of data processing electronics. The exploration of solutions based on self-assembled elemental blocks to mimic biological networks’ complexity is explored in the general frame of unconventional computing and it has not reached yet a maturity stage enabling a benchmark with standard electronic approaches in terms of performances, compatibility and scalability. Here we discuss some aspects related to advantages and disadvantages in the emulation of the brain for neuromorphic hardware. We also discuss possible directions in terms of hybrid hardware solutions where self-assembled substrates coexist and integrate with conventional electronics in view of neuromorphic architectures.

## 1 Introduction

The reduction of the energy footprint of pervasive computing infrastructures is crucial for the realization of a sustainable innovation agenda (Masanet et al., 2020). The advent of artificial intelligence (AI) has made even more urgent the development of suitable computing technologies to try to contain the enormous expenditure of energy required for data storage and processing infrastructures (Masanet et al., 2020; De Vries, 2023).

AI achievements in areas such as speech and visual object recognition, object detection, and various other fields within hard and life sciences can be attributed to the use of deep learning techniques that utilize computational models made up of multiple processing layers, enabling them to learn data representations with varying levels of abstraction (Kozma et al., 2018). Deep Neural Networks (DNN) effectively identify complex patterns within large datasets, as Spiking Neural Network (SNN) in vision and event-based tasks, while Recurrent Neural Networks (RNN) are used for complex sequential data like text and speech series (LeCun et al., 2015). These methods exploit at different levels gradient descent via backpropagation algorithm and the highly parallel matrix multiplications enabled by GPUs (Burr et al., 2017). Recently, Transformer architectures have revolutionized AI by overcoming some limitations of previous models like RNNs, particularly in sequential tasks. Transformers, which rely on self-attention mechanisms, have demonstrated superior performance in language models (Vaswani et al., 2017) and, more recently, Vision Transformers (ViTs) have extended these principles to computer vision tasks (Dosovitskiy et al., 2021). Unlike Convolutional Neural Networks (CNNs), which focus on local patterns, ViTs can capture global context efficiently, further pushing the boundaries of AI applications, but often at the cost of higher energy consumption due to their computational complexity.

To reverse the trend of increasingly growing energy demand of DNN, the use of new biologically inspired strategies for computing and data processing is actively pursued (Aimone, 2021). Human brain is considered the essential model of the matching of energy efficiency and structural complexity with dynamic learning capabilities in the context of unstructured noisy data (Axer and Amunts, 2022). The ability of the brain to perform such efficient and fault-tolerant data processing is strongly related to its peculiar interconnected adaptive architecture, based on redundant multiscale neural circuits and equipped with short- and long-term plasticity (Sporns et al., 2005; Shapson-Coe et al., 2024). During evolution, animals have faced the constant problem of energy scarcity: to afford a metabolically expensive brain, they have evolved finding strategies for the implementation of energy-efficient neural coding, enabling operation at reduced energy costs (Padamsey and Rochefort, 2023).

The intimate interrelation between multiscale structure and function in the brain is a fundamental aspect, still poorly understood (Breakspear and Stam, 2005; Suárez et al., 2020), for the optimization of internal and external resources: as an example, one can consider that information transmission through presynaptic terminals and postsynaptic spines is related to their energy consumption (Harris et al., 2012; Padamsey and Rochefort, 2023). Although conspicuous intellectual and economic efforts have been concentrated in the last decades on the artificial reproduction of the human brain performances in terms of energetic resource optimization, precision and timeliness, this achievement remains a challenge yet to be overcome (Pfaff et al., 2022; Shapson-Coe et al., 2024).

Neuromorphic engineering and computing (Mead, 1990) aim at the implementation of highly efficient information processing methods typical of biological systems on artificial software and hardware platforms, relying on the tacit assumption that the brain functions like a computer and that a computer can function like the brain (Jaeger, 2021; Richards and Lillicrap, 2022). Since Alan Turing’s groundbreaking work on computability (Turing, 1937), the concept of “computable functions” has been central to define what computers can achieve, regardless of their physical characteristics. While a brain could potentially emulate a Turing machine in terms of its computational capabilities, it operates in a fundamentally different way from traditional computers (Jaeger, 2021; Jaeger et al., 2023). Furthermore, in computers distinct boundaries exist between the physical hardware and the software algorithms that run on them, while in the brain the categories of hardware and software have no practical meaning (Jaeger et al., 2023).

The dichotomy between hardware and software is probably one of the major obstacles to the realization of an artificial system truly neuromorphic and it profoundly affects the way artificial neuromorphic systems are conceived and perceived (Jaeger, 2021). DNN are considered as brain-inspired since they are organized on several layers of artificial neurons and synapses (Haensch et al., 2023), however despite this terminology is derived from the neurosciences, the actual correspondence to the main mechanisms of the natural neural counterparts is far from being implemented and their training approaches do not represent the learning strategy adopted by the biological system (Burr et al., 2017).

On the hardware side, major efforts are concentrated on solutions that can improve the energy efficiency of DNNs (Chen et al., 2022). Among them the use, at various length scales, of building blocks and/or architectures reproducing biological brain components and organization is gaining increasing attention (Burr et al., 2017). In particular, advances in neuromorphic hardware addresses two major distinctive features of the biological brain data processing: the temporal dimension in data transmission (Spiking Neural Networks) (Schuman et al., 2022) and the so-called in memory computing (Burr et al., 2017). Strategic hardware components for the implementation of efficient SNN are dense crossbar arrays of non-volatile memory (NVM) devices as an alternative to CMOS neuron circuitry. These could represent an effective solution for the improvement of energy efficiency although they have a very poor similarity to biologic neurons and synapses. The faithful imitation of the structural and functional characteristics of biological constituents is not in itself a necessary and sufficient condition for the substantial increase in performance of neuromorphic software based on DNN.

Considering the brain on larger scales compared to that typical of a single or small groups of neurons and synapses, one can affirm that the structural and functional properties in terms of adaptability, learning capability, robustness and efficiency are related to its self-organized nature (Kelso, 2012). Self-organization achieves stability and functional plasticity while minimizing structural system complexity, it can be defined in terms of a general principle of functional organization that ensures system autoregulation, adaptation to new constraints, and functional autonomy (Dresp-Langley, 2020). These characteristics can be recognized in many natural dynamic and adaptive systems based on organic and inorganic matter and capable of evolving in response to internal and external stimuli (Tanaka et al., 2019). It seems thus reasonable to hypothesize that systems different from the biological brain and characterized by self-organization can be considered for computation and data processing.

Such systems are at the basis of the so-called unconventional computing approach (Finocchio et al., 2024; Vahl et al., 2024), where computation arises from the collective interactions of many simple components and by the emergence of complex patterns rather than from a central processing unit communicating with memory. It would be interesting to discuss the reasons why the term “unconventional” is used in this context, instead of neuromorphic. In our opinion, one of the reasons is linked to the very strong significance of the brain-computer metaphor. The use of features related to self-assembly and statistical mechanisms for data processing, even if typical of the biological brain, are not perceived as “neuromorphic” as they are very far from the architectures of a traditional computer. Anyway, by using materials having memory effects and nonlinear responses, it could be possible to create devices performing “neuromorphic” tasks like pattern recognition or time-series analysis with low external energy input (Vahl et al., 2024), being based on the spontaneous reorganization of the physical system. Such data processing can be performed through various physical phenomena, such as electrical conductivity, magnetism, and even chemical reactions (Vahl et al., 2024).

Networks of nano-objects electrically connected by junctions possessing nonlinear memristive characteristics (Milano et al., 2023) exhibit emergent complexity and collective phenomena akin to biological neural networks, in particular hierarchical collective dynamics (Mallinson et al., 2019) and heterosynaptic plasticity (Fostner and Brown, 2015; Milano et al., 2020, 2022a; Daniels and Brown, 2021; Kuncic and Nakayama, 2021; Carstens et al., 2022; Vahl et al., 2024). Data processing performed with these nanostructured systems has been reported under the label of “in materia computing”(Jaeger, 2021; Milano et al., 2022b). The emerging properties of self-assembled networks of nanowires and nanoparticles have been used to provide a physical substrate for Reservoir Computing (RC) (Usami et al., 2021; Daniels et al., 2022; Milano et al., 2022b; Yan et al., 2024). RC is a paradigm in machine learning that utilizes a fixed, complex dynamic system as a “reservoir” to process input signals (Miller, 2019; Tanaka et al., 2019; Nakajima, 2020).

The exploitation of the in materia approach is in its infancy and it must face different challenges: a major one, common to all the unconventional approaches to computation, is represented by the lack of a unifying theoretical basis, as discussed in detail in Jaeger (2021). The second is the benchmarking in terms of material selection, computing performances, fabrication scalability with the silicon-based technology (classical and/or neuromorphic) (Teuscher, 2014; Cao et al., 2023).

Here we would like to address some issues related to the possibility of using hardware solutions proposed for in materia computing and mimicking the biological brain as a self-assembled system, for tasks demanded to components and architectures typical of neuromorphic or even standard von Neumann systems. The development of real devices based on in materia computing systems can provide useful insights for the translation of a “theory of computing from whatever physics offer” (Jaeger et al., 2023) to an effective hardware. To analyze this point, we will consider some aspects of the picture describing the biological brain related to nonlinearity and nonlocality, then we will discuss possible artificial counterparts and their integration for the fabrication of devices for Boolean functions classification.

## 2 Biological picture

The operation of mammalian brains relies on optimizing and organizing a multitude of biochemical, electrophysiological, and anatomical phenomena across various spatial and temporal scales, culminating in an incredibly adaptive, robust, and balanced physical system (Herculano-Houzel, 2009; Loomba et al., 2022). Key building blocks include cells such as neurons, glial cells, and astrocytes, which intricately connect and through synapses, form complex functional networks (Shapson-Coe et al., 2024). Chemical synapses are prevalent in the brain and involve several sequential steps, starting with the transmission of an action potential through the axon, leading to neurotransmitters release in the synaptic cleft. This is followed by neurotransmitters binding to post-synaptic receptors, ultimately activating an ionic current in the post-synaptic cell. The multipart process of generating a postsynaptic current is subject to various modulation and plasticity phenomena, influencing information processing within neurons and neural circuits (Stöckel and Eliasmith, 2021).

Understanding changes in synaptic strength has been a focus of research, utilizing different stimulation protocols to assess their impact on computation. Short-term plasticity (STP) acts as a band-pass filter for incoming signals (Tsodyks and Wu, 2013), while long-term potentiation/depression (LTP/LTD) results in a lasting alteration of synaptic strength, crucial for learning and memory (Xu-Friedman and Regehr, 2003; Nieus et al., 2006). The interplay between different plasticity phenomena is noteworthy, with studies demonstrating how LTP/LTD dynamics can turn synaptic facilitation into depression, influencing how neural cells process input stimuli (Arleo et al., 2010).

**Figure 1:**
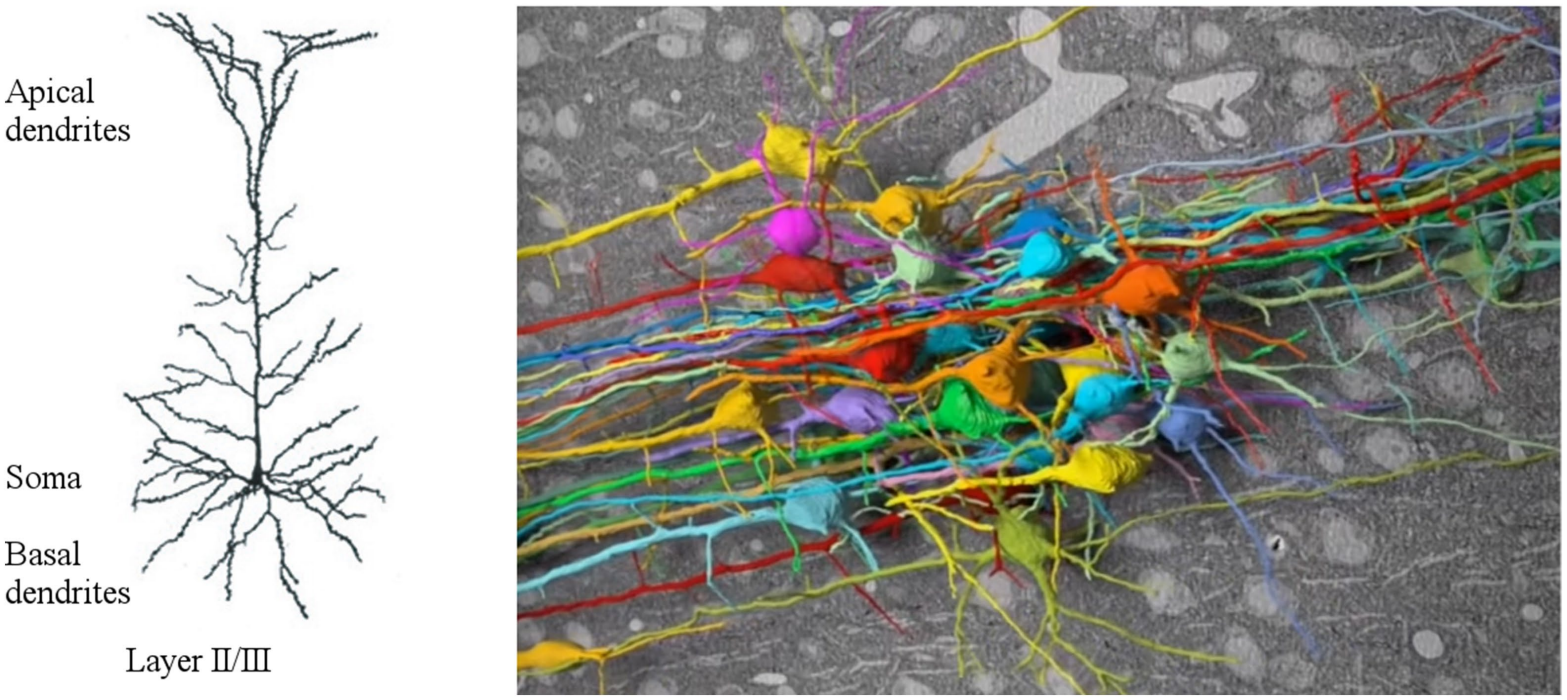
On the left, dendritic tree of pyramidal neurons from cortical layer 2 and 3, featured by two distinct domains, i.e., the basal and the apical dendrites, adapted from Spruston (2008); on the right, connectome from human brain, adapted from Carving Out Brain Structure with Connectomics (2022).

Furthermore, neurons are structurally extended, with dendritic trees spanning up to 1 mm, which significantly impacts a neuron’s computational abilities. For example, cortical layer 5 pyramidal neurons (examples of pyramidal neurons from cortex area are shown in Figure 1) function as coincidence detectors for contextual and sensory inputs, playing a crucial role in connecting cortical columns and the thalamus, believed to be fundamental for consciousness (Aru et al., 2019). Over the past three decades, extensive research into cortical pyramidal neurons has unveiled a multitude of their processing capabilities (Spruston, 2008). Recent findings have even demonstrated that in human cortical layer 2 and 3 pyramidal neurons can solve the XOR operation (Gidon et al., 2020), a task typically achieved only by multilayer artificial neural networks.

Thus, the computational capacity of a neural ensemble (a group of neurons performing a specific function) appears to be enhanced not only by the interconnections within the recurrent network (a portion of human brain’s connectome is shown in Figure 1) but also by the dynamic nature of neurons themselves, particularly through their intricate processing of inputs at the dendritic level (Häusser et al., 2000). Studies have shown that back-propagating action potentials interact with synaptic inputs, amplifying dendritic calcium signals nonlinearly and increasing neuron firing rates (Larkum et al., 1999). Nonlinear systems can embody various types of computations, and it is feasible to dynamically configure the system to execute different ones (Kia et al., 2017). The adaptability of living systems itself to diverse conditions is believed to stem from nonlinearity (Skarda and Freeman, 1987).

Biophysically plausible computational models suggest that the nonlinear properties of dendrites are crucial, for example in explaining the processing capabilities of cortical pyramidal neurons. Nonlinear phenomena can arise from the interaction between the spatial distribution of ionic channels and synapses (Mäki-Marttunen and Mäki-Marttunen, 2022). The arrangement of synapses and ionic channels in dendritic trees plays a fundamental role for the understanding of how neurons contribute to information processing (Migliore and Shepherd, 2002). Activation of the NMDA synaptic receptor exhibits high nonlinearity, akin to a transistor’s response concerning the post-synaptic site’s voltage. Moreover, under specific conditions, NMDA receptor activation can trigger a series of intracellular processes involved in long-term potentiation (LTP) formation (Luscher and Malenka, 2012). Furthermore, compartmental models of passive dendrites demonstrate that adjacent synapse activations tend to sum less linearly compared to distant synapses, which tend to sum linearly. This spatial sensitivity implies that local nonlinear synaptic operations can be semi-independently executed in numerous dendritic subunits (Kia et al., 2017).

An original attempt to insert non linearity in modeling dendrites inputs integration is reported in Legenstein and Maass (2011), where the summing of post-synaptic potentials at dendritic branches is modeled as a weighted linear combination of input potentials (passive terms) and active nonlinear components, which activate when passive elements exceed a specific dendritic threshold. Here, nonlinearity is effectively integrated into dendritic dynamics, although input-associated weights are treated independently.

Also at a larger scale, the nervous system adjusts its functioning through nonlinear changes in activation patterns within networks of cells composed of large numbers of units; these networks are responsible for channeling, shifting, and shunting activity (Kozachkov et al., 2020). The activity of the biological network itself emerges as significant output, where synchronized neural activities and feedback loops are key elements of its operating mechanisms. Neurons respond in an analog way, changing their activity in response to changes in stimulation. Unlike any artificial device, the nodes of these networks are not stable points like transistors or valves, but sets of neurons—hundreds, thousands, tens of thousands strong—that can respond consistently as a network over time, even if the component cells show inconsistent behavior (Richards and Lillicrap, 2022).

The organization of brain connectivity, by a very simple level of single neuron dendritic tree to more complex neuronal ensemble, is at the basis of remarkable properties such as fault tolerance, robustness and redundancy (Kitano, 2004).

The brain is characterized by robustness, being able to support highly efficient information transmission between neurons, circuits, and large regions making it possible to promptly gather and distribute information while tolerate the large-scale destruction of neurons or synaptic connections (Achard et al., 2006; Tian and Sun, 2022). Till now, it remains unclear where these remarkable properties of brain originate from. The close relationships between these properties and the brain network naturally leads to a hypothesis that argues these properties may originate from specific characteristics of brain connectivity (Tian and Sun, 2022).

## 3 Artificial brain-like hardware

### 3.1 Building blocks

The theoretical underpinnings of the artificial neuromorphic primitive elements are rooted in modeling biological neuronal systems as propositional logic units, as proposed by McCulloch and Pitts (1943). They suggested that due to the “all-or-none” nature of nervous activity, neural events and their relations can be handled using propositional logic. Subsequently, Donald Hebb’s findings on neural network plasticity led to a deeper exploration and formalization of constituent elements, as networks exhibit learning abilities and reinforce connections (Paulsen, 2000). In 1952, John von Neumann postulated that logical propositions can be represented as electrical networks or idealized nervous systems, setting the foundation for current computation models (Neumann, 1956). An aspect that seems to be scarcely recognized is that the above-mentioned models are mainly used to build neuromorphic software, and they are only marginally translated into hardware.

Threshold logic gates (TLGs) are directly inspired to the neuron (McCulloch and Pitts picture) and constitute the basis of many software neuromorphic architectures, however they are not used in conventional and neuromorphic hardware. Digital circuit design is entirely based on Boolean logic circuits and not on TLGs, although the ability of the latter to process multiple inputs simultaneously and perform weighted calculations for efficient implementation of complex functions (Beiu et al., 2003). TLGs also provide natural fault tolerance and are more resilient to noise, given their analog-based approach to digital computation (Zhang et al., 2008).

Boolean logic circuits are widespread because of their simplicity and easiness to design, debug, and manufacture (Elahi, 2022). The vast existing literature and experience in Boolean logic facilitate rapid prototyping and scalability, making them the industry standard for von Neumann and neuromorphic computing applications. Additionally, they can take advantage of established fabrication technologies, leading to cost-effective manufacturing. The simplicity of Boolean circuits has led to their standardization across the industry, with established design methodologies, tools, and educational resources readily available (Vingron, 2024). CMOS technology is designed around Boolean logic gates for mass production in consumer electronics, computing devices, and embedded systems.

We can summarize that brain-inspired building blocks such as threshold logic gates (neuron) and input weights (synapses) are at the basis of neuromorphic software running on a hardware, where Boolean logic gates are organized in order to improve the efficiency of the neuromorphic software. The development of neuromorphic computing hardware by using CMOS architectures (Indiveri et al., 2011) or other types of devices such as non-volatile memories (Indiveri et al., 2013; Burr et al., 2017; Ielmini and Ambrogio, 2020) to emulate neurobiological networks at the small circuit or device level, may favor a significant reduction of power consumption (Li et al., 2021). The downscaling of dense non-volatile memory (NVM) crossbar arrays to few-nanometer critical dimensions has been recognized as one path to build computing systems that can mimic the massive parallelism and low-power operation found in the human brain.

### 3.2 Brain-like networks

**Figure 2:**
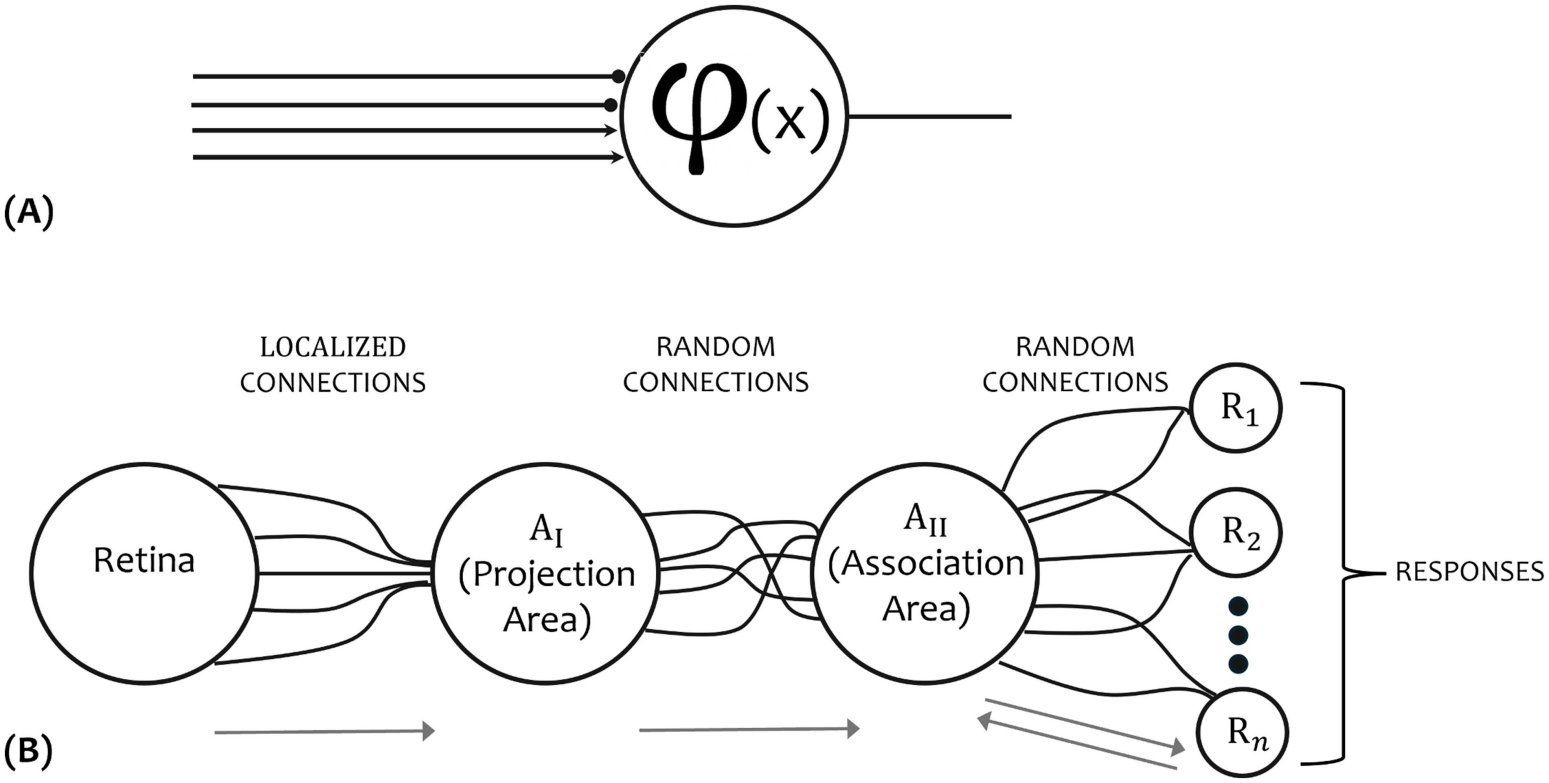
Neuron model proposed by Neumann (1956) **(A)**, and the organization of the perceptron proposed by Rosenblatt (1958) **(B)**.

The fundamental significance of the structural organization of biological systems was recognized by F. Rosenblatt, who introduced in a series of seminal works the idea of weighted sums of inputs and network reinforcement in his perceptron model (Rosenblatt, 1958). This model was not primarily concerned with the invention of a device for “artificial intelligence,” but rather with investigating the physical structures and neurodynamic principles which underlie “natural intelligence” (Rosenblatt, 1962). The perceptron was considered a brain model, not a device for pattern recognition; as a brain model, its utility was concentrated in enabling to determine the physical conditions for the emergence of various psychological properties (Rosenblatt, 1962) (Figure 2B).

In his probabilistic theory, he emphasized statistical separability as the core of biological intelligence, rather than symbolic logic (Rosenblatt, 1958). His model had significant “neuromorphic” aspects compared to those based solely on McCulloch and Pitts neurons (Figure 2A), particularly in the role of connections, both random and strategically placed within the projected area, such as in the photo-perceptron organization, and the reinforcement system (Figure 2B). Positive and negative reinforcement play key roles in either facilitating or hindering the reorganization of connections, whereas McCulloch and Pitts networks were assumed to be fixed (McCulloch and Pitts, 1943).

In Rosenblatt’s model, complexity arises from the extensive number of interconnections rather than the variety of basic components (Rosenblatt, 1958). He proposed the concept of combining wiring, specifically random wiring, as crucial for information storage, in analogy with biological system, so much so that the model was specifically developed to address questions regarding information storage and its influence on brain recognition and behavior.

Rosenblatt also underlined that “…The construction of physical perceptron models of significant size and complexity is currently limited by two technological problems: the design of a cheap, mass-produceable integrator, and the development of an inexpensive means of wiring large networks of components” (Rosenblatt, 1962).

The importance of random wiring pointed out by Rosenblatt was not fully recognized (Rosenblatt, 1958) and the perceptron was considered as a threshold logic gate able to perform linear classification. The linear character of the device is a limitation, as pointed out by Minsky and Papert (2017) and nonlinearity can be obtained only by the use of perceptron networks (Hopfield, 1982; Cybenko, 1989; Olivieri, 2024).

## 4 Self-assembled brain-like hardware

Among the materials which offer a degree of structural and functional complexity at different scales, self-assembled networks of nanoobjects connected by nonlinear electric junctions are characterized by complex and redundant wiring (Mirigliano and Milani, 2021; Vahl et al., 2024). Compared to standard circuits typical of conventional computing systems, they are much easier to assemble, however quite intractable in terms of designing, interfacing, and interconnecting with conventional computing hardware (Vahl et al., 2024).

Recently, a logic threshold gate has been proposed based on self-assembled nanostructured films that share some of the characteristics of random wiring and reconfigurability typical of biological systems and present in the original Rosenblatt’s perceptron model. This model, called “Receptron” (reservoir perceptron), share with Rosenblatt’s perceptron the fundamental importance of the randomness of the connections, on the other hand it generalizes the Minsky perceptron considered as a linear logic gate by introducing, as in the biological neuron, a nonlinear dependence among the input weights (Martini et al., 2022; Paroli et al., 2023). The Receptron instead is a reconfigurable, non-linear threshold logic gate (Mirigliano et al., 2021; Martini et al., 2022; Paroli et al., 2023).

From a formal standpoint, one can consider the traditional logic threshold perceptron model as based on linearly independent weights:


(1) $$S=\overset{n}{\underset{j=1}{\sum }}x_{j}w_{j}\text{,}$$

where j numbers the inputs ( $j\in \left[1,n\right]$ ) and $w_{j}$ are constant real values referring to the weights in the perceptron model.

A more general form of Equation 1 can be considered which allows for the nonlinear interaction of the inputs:


(2) $$S=\overset{n}{\underset{j=1}{\sum }}x_{j}\tilde{w_{j}}\left(\vec{x}\right)\;|\;S\in R\text{,}$$

where $\tilde{w_{j}}\left(\vec{x}\right):R^{n}\to C$ are complex-valued functions and $\vec{x}=\left(x_{1},\ldots ,x_{n}\right)$ is the input vector (Paroli et al., 2023).

Equation 2 is at the basis of the Receptron formalism (see Paroli et al., 2023) and it describes a threshold system where the input weights are not univocally related to a single input, hence, they cannot be independently adjusted. This nonlinear characteristic is similar to what observed in the interactions between synapses in neural dendritic trees (Moldwin and Segev, 2020). In this sense the model of the receptron is a generalization of that of the perceptron as a logic threshold gate: the weights are not univocally related to a single input making the Receptron intrinsically nonlinear and capable, as a single device, of classification tasks not achievable by individual perceptrons (Paroli et al., 2023).

Physical systems fabricated by the random assembly of metallic nanoparticle from the gas phase to form nanostructured films constitute a suitable hardware to implement the Receptron, since they consist in a network of highly interconnected non-linear junctions regulating their connectivity and the topology of conducting pathways depending on the input stimuli (Mirigliano et al., 2020; Martini et al., 2024). Two-electrodes and multi-electrodes planar devices based on cluster-assembled Au and Pt showing nonlinear electronic properties and resistive switching behavior have been fabricated by supersonic cluster beam deposition (SCBD) (Mirigliano and Milani, 2021; Radice et al., 2024).

**Figure 3:**
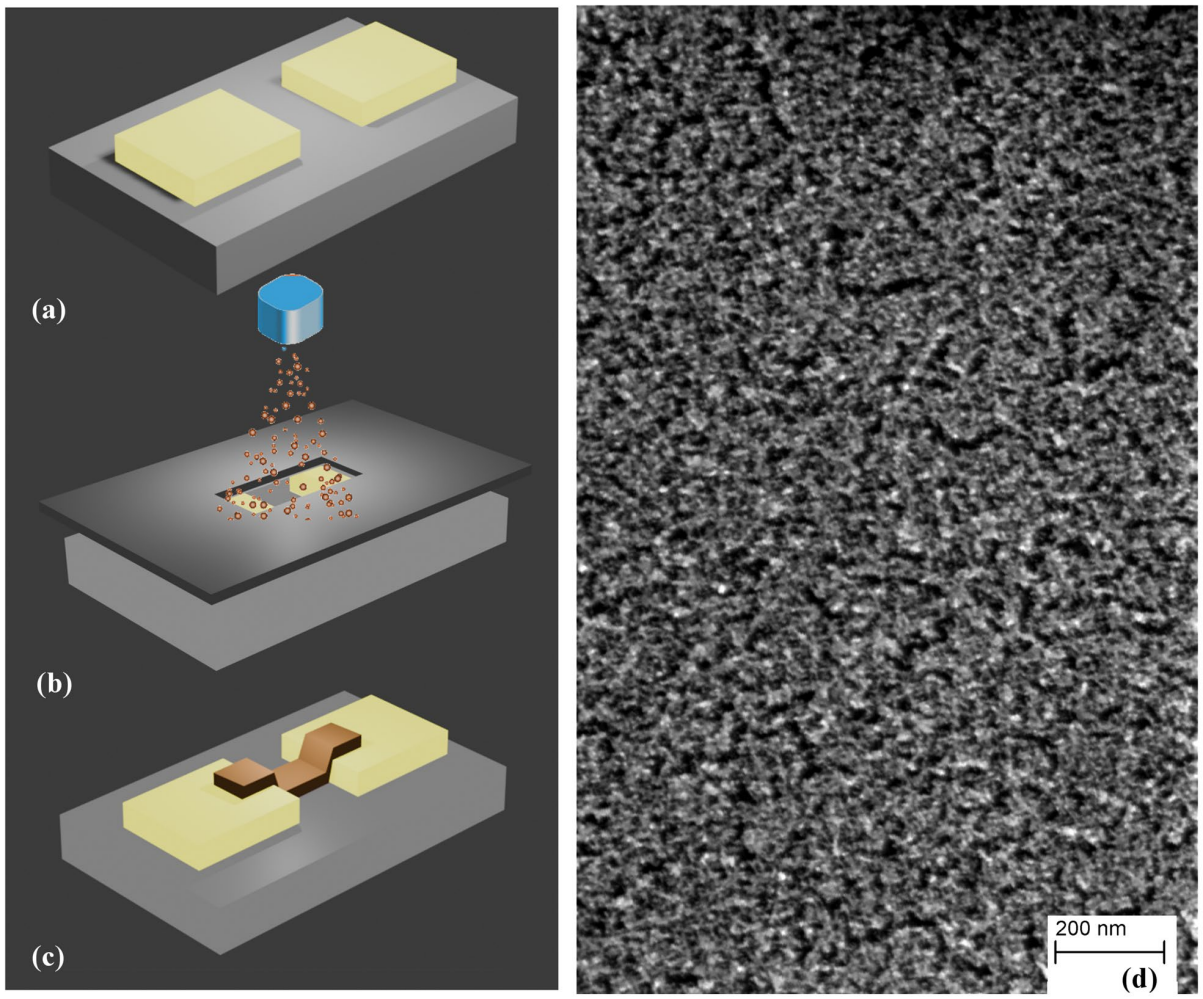
Schematic representation of cluster-assembled thin film deposition: **(A)** two or multi metallic electrodes are deposited on a flat and insulating substrate, **(B)** a mask is placed between the clusters beam and the sample for its negative printing on the substrate, **(C)** the mask is removed and a nanostructured film with rough and disordered structure **(D)** is formed.

SCBD is a well-established technology for the scalable production of nanostructured metallic and oxide thin films (Borghi et al., 2018, 2019, 2022; Profumo et al., 2023; Radice et al., 2024) characterized by a high deposition rate, high lateral resolution (compatible with planar microfabrication technologies) and neutral particle mass selection process by exploiting aerodynamic focusing effects (Tafreshi et al., 2001; Barborini et al., 2003). High directionality, collimation and intensity of aerodynamically focused supersonic cluster beams, make it suitable for patterned deposition of nanostructured films through non-contact stencil masks or lift-off technologies (Previdi et al., 2021), as schematically described in Figure 3, enabling this tool for the large-scale integration of nanoparticles and nanostructured films on microfabricated platforms and smart nanocomposites (Marelli et al., 2011; Migliorini et al., 2020, 2021, 2023).

SCBD can be used for high throughput fabrication of two-electrode and multielectrode nanostructured metallic planar devices characterized by resistive switching behavior (Borghi et al., 2022; Martini et al., 2022; Profumo et al., 2023; Radice et al., 2024). Due to the efficient decoupling of cluster production, manipulation and deposition in a typical SCBD apparatus it is possible to characterize *in situ* the evolution of the electrical properties of cluster-assembled films during the fabrication process. This allows the precise and reproducible production of large batches of films with tailored electrical properties (Mirigliano et al., 2020; Mirigliano and Milani, 2021).

In particular, nanostructured Au and Pt films fabricated by supersonic cluster beam deposition (Mirigliano et al., 2019, 2020; Radice et al., 2024) show a complex resistive switching behavior and their nonlinear electric conduction properties are deeply affected by the extremely high density of grain boundaries resulting in a complex network of nanojunctions (Mirigliano et al., 2019, 2020; Nadalini et al., 2023; Profumo et al., 2023; Radice et al., 2024). Correlations emerge among the electrical activity of different regions of the film under the application of an external electrical stimulus higher than a suitable threshold. The degree of correlation can be varied controlling the film connectivity at the nano- and mesoscale (Nadalini et al., 2023), as also its geometry and the electrode configuration used as input and output (Martini et al., 2022, 2024).

A possible hardware implementation of a Receptron has thus been obtained by interconnecting a generic pattern of electrodes with a cluster-assembled Au film; this multielectrode device can perform the binary classification of input signals, following a thresholding process, to generate a set of Boolean functions (Mirigliano et al., 2021). The multielectrode Receptron can receive binary inputs from all the possible combination of the input electrodes and generate a complete set of Boolean functions of n variables for classification tasks.

**Figure 4:**
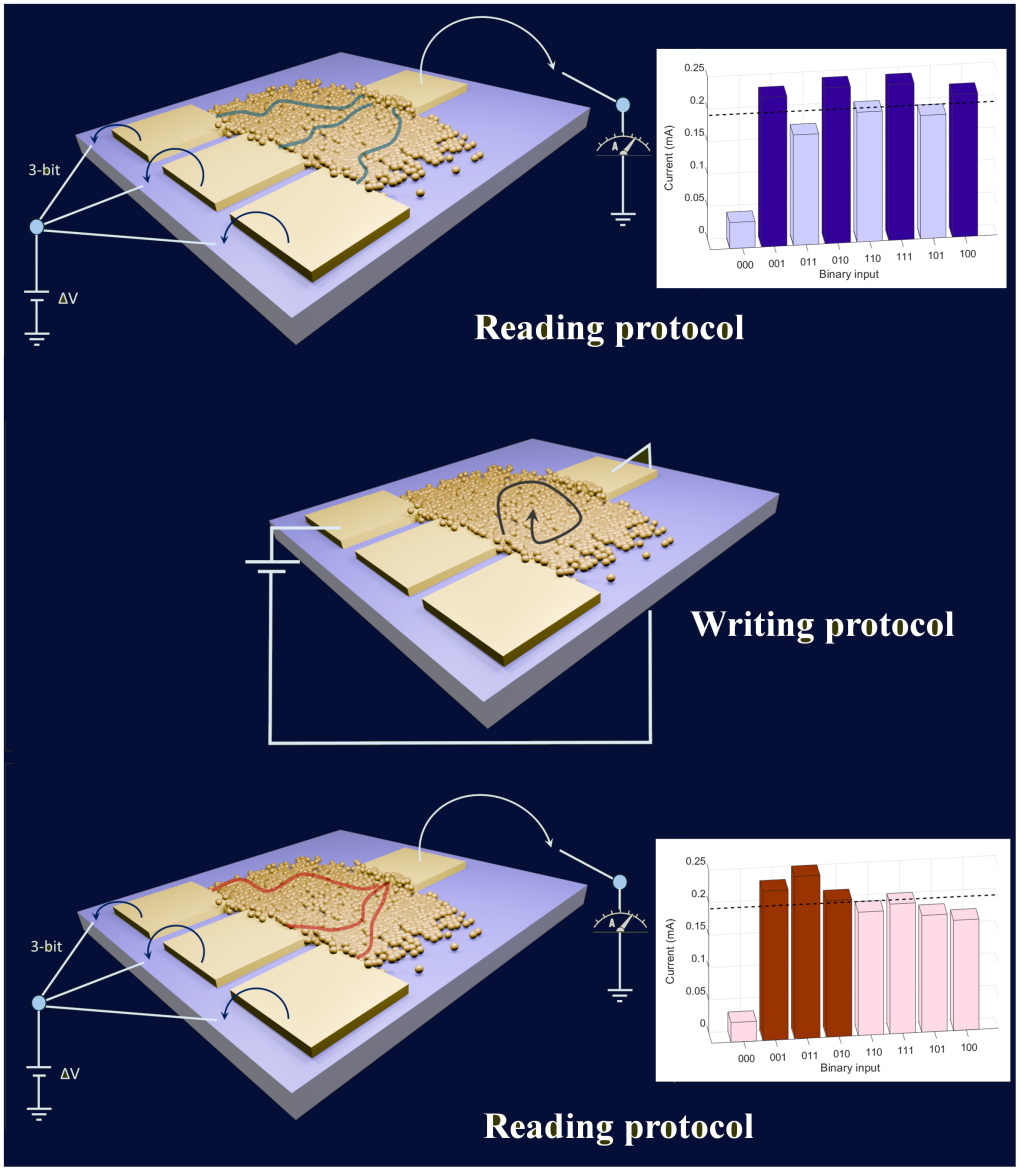
Schematic representation of multielectrode device based on nanostructured random-assembled material, implementing a Boolean functions classifier according to the Receptron model. The Boolean function is obtained by thresholding the analog outputs recorded at low voltage (lower than 1 V) for all the possible combinations of the 3-bit inputs. By applying a short pulse of a voltage highest than a certain threshold between a pair of electrodes, the connectivity of the network changes and a new conductance map is written. After this stochastic writing process, a new sequence of analog outputs is recorded, by implementing a different Boolean function.

In analogy with neural biological systems, the network of interconnected nanojunctions are characterized by the nonlinear and distributed nature of the junction weight interactions: the weights are not exclusively tied to a single node as highly interconnected junctions modulate their connectivity and conducting pathways’ topology based on input stimuli (Mirigliano et al., 2020, 2021; Martini et al., 2022), mirroring the behavior observed in neuronal dendrites (Moldwin and Segev, 2020; Bicknell and Häusser, 2021). The device can switch between reconfiguration and computation functionalities. The reading of the analog output for each combination of a 3-bit system is performed at low voltage, not to change the resistivity map of the gold network (reading process in Figure 4), to compute a specific Boolean function. By exploring the resistive switching behavior of the nanogranular material, by means of an electrical stimulus applied to different possible pairs of electrodes (writing procedure in Figure 4), the electrical response of the network changes in a nonlinear and nonlocal fashion (Martini et al., 2022), allowing the reconfiguration of the device. It is thus possible to generate an extremely wide range of outputs, which change the sequence of their analog values (Martini et al., 2022). The outputs cannot be directly and accurately controlled: because of the extremely high number of junctions involved and the metastable configurations of the system, thus making receptron a kind of random and unpredictable device. Therefore, one could not exploit this device in a pre-programmed and deterministic way: one can instead use this complex system to rapidly explore a variety of input–output applications until the right one is reached (Martini et al., 2022).

A schematic representation of the electrical setup for a 3-bit receptron device is reported in Figure 4: it is featured by three relays on the left, which are connected to their respective electrodes, to enable switching between a voltage supply and an open circuit, on the right, the output electrode is connected to a digital multimeter, allowing for the current recording.

In a 3-bit receptron all 2^3^ = 8 possible input combinations are tested, each producing a corresponding output that can be digitized through thresholding, thus generating a Boolean function (see Figure 4). Subsequent reconfiguration alters the nanostructure of the gold film, potentially modifying the preferred input–output current pathways through the formation or disruption of grain boundaries and other defects, generating a new function.

This random search method is intrinsically different from the training performed for classical DNNs: while classical neural networks require fine tuning of the independent weights of each single node according to some gradient descent technique (Rumelhart et al., 1986; Huang et al., 2020), the Receptron approach relies only on a random change of the interconnected weights (Mirigliano et al., 2021; Martini et al., 2022). The non-local resistive switching behavior in the nanojunction system result in a tunable correlated behavior, characterized by a non-trivial simultaneous changes in the resistivity of different regions of the film, generating voltage outputs which are not statistically independent (Martini et al., 2022).

The using of a stochastic approach for Boolean function classification with a nanostructured device fabricated by the self-assembling of metallic nanoparticles might unconsciously suggest that one is dealing with an irreproducible and unstable system. This is not the case: the stability and reproducibility of the Receptron device has been tested over period of several months and in certain cases of years in normal laboratory conditions. A reconfigurable arithmetic logic unit based on Receptrons instead of Boolean circuits is currently underway (Martini, n.d.).

## 5 Summary

Neuromorphic computing refers to a wide variety of software/hardware architectures and solutions that try to emulate the levels of energy efficiency and data processing performance typical of the biological brain. The strategies adopted and the terminologies used are affected on one hand by the tendency to consider computers and brains as ideally superimposable and, on the other hand, by the unavoidable dichotomy between hardware and software that characterizes artificial systems based on Turing and von Neumann paradigms (Jaeger, 2021; Jaeger et al., 2023).

Neuromorphic computing software is exploiting hardware platforms based on highly miniaturized and integrated Boolean logic gate architectures and not on threshold logic gates which are considered the electronic counterpart of McCulloch and Pitts neurons. The hardware of neuromorphic systems is currently based on top-down fabrication approaches and not on self-assembling and redundant wiring typical of biological neural systems.

The use of self-assembled substrates for computing, although characterized by features like those of biological systems, is considered as “unconventional.” Although interesting results have been reported in the case of reservoir computing using self-assembled systems, strategical issues about scalability, reproducibility and compatibility with CMOS architectures are still to be addressed. A possible route in this direction is the use of non-linear threshold logic gates based on self-assembled nanostructured substrates (receptrons) to build devices that can be integrated with standard electronic systems.

Solutions based on truly brain-like hardware capable of substituting CMOS architectures for neuromorphic computing are not on the horizon so far and realistically will not be for a long time to come (Teuscher, 2014). Unconventional and CMOS hardware should find a mutually profitable coexistence regardless of the faithful reproduction of the biological neural structures. To this goal the development of reconfigurable threshold logic gates based on self-assembled nanostructured substrates that can be integrated in standard microelectronic architectures can be considered as an interesting starting point for the further development of hybrid computing hardware.

## Data Availability

The raw data supporting the conclusions of this article will be made available by the authors, without undue reservation.

## References

[ref1] Achard S. Salvador R. Whitcher B. Suckling J. Bullmore E. (2006). A resilient, low-frequency, small-world human brain functional network with highly connected association cortical hubs. J. Neurosci. 26, 63–72. doi: 10.1523/JNEUROSCI.3874-05.2006, PMID: 16399673 PMC6674299

[ref2] Aimone J. B. (2021). A roadmap for reaching the potential of brain-derived computing. Adv. Intell. Syst. 3:2000191. doi: 10.1002/aisy.202000191

[ref3] Arleo A. Nieus T. Bezzi M. D’Errico A. D’Angelo E. Coenen O. J.-M. D. (2010). How synaptic release probability shapes neuronal transmission: information-theoretic analysis in a cerebellar granule cell. Neural Comput. 22, 2031–2058. doi: 10.1162/NECO_a_00006-Arleo, PMID: 20438336

[ref4] Aru J. Suzuki M. Rutiku R. Larkum M. E. Bachmann T. (2019). Coupling the state and contents of consciousness. Front. Syst. Neurosci. 13:43. doi: 10.3389/fnsys.2019.00043, PMID: 31543762 PMC6729974

[ref5] Axer M. Amunts K. (2022). Scale matters: the nested human connectome. Science 378, 500–504. doi: 10.1126/science.abq2599, PMID: 36378967

[ref6] Barborini E. Kholmanov I. N. Conti A. M. Piseri P. Vinati S. Milani P.. (2003). Supersonic cluster beam deposition of nanostructured titania. Eur. Phys. J. D 24, 277–282. doi: 10.1140/epjd/e2003-00189-2

[ref7] Beiu V. Quintana J. M. Avedillo M. J. (2003). VLSI implementations of threshold logic- a comprehensive survey. IEEE Trans. Neural Netw. 14, 1217–1243. doi: 10.1109/TNN.2003.816365, PMID: 18244573

[ref8] Bicknell B. A. Häusser M. (2021). A synaptic learning rule for exploiting nonlinear dendritic computation. Neuron 109, 4001–4017.e10. doi: 10.1016/j.neuron.2021.09.044, PMID: 34715026 PMC8691952

[ref9] Borghi F. Milani M. Bettini L. G. Podestà A. Milani P. (2019). Quantitative characterization of the interfacial morphology and bulk porosity of nanoporous cluster-assembled carbon thin films. Appl. Surf. Sci. 479, 395–402. doi: 10.1016/j.apsusc.2019.02.066

[ref10] Borghi F. Mirigliano M. Dellasega D. Milani P. (2022). Influence of the nanostructure on the electric transport properties of resistive switching cluster-assembled gold films. Appl. Surf. Sci. 582:152485. doi: 10.1016/j.apsusc.2022.152485

[ref11] Borghi F. Podestà A. Piazzoni C. Milani P. (2018). Growth mechanism of cluster-assembled surfaces: from submonolayer to thin-film regime. Phys. Rev. Appl. 9:044016. doi: 10.1103/PhysRevApplied.9.044016

[ref12] Breakspear M. Stam C. J. (2005). Dynamics of a neural system with a multiscale architecture. Philos. Trans. R. Soc. B Biol. Sci. 360, 1051–1074. doi: 10.1098/rstb.2005.1643, PMID: 16087448 PMC1854927

[ref13] Burr G. W. Shelby R. M. Sebastian A. Kim S. Kim S. Sidler S.. (2017). Neuromorphic computing using non-volatile memory. Adv. Phys. X 2, 89–124. doi: 10.1080/23746149.2016.1259585

[ref14] Cao W. Bu H. Vinet M. Cao M. Takagi S. Hwang S.. (2023). The future transistors. Nature 620, 501–515. doi: 10.1038/s41586-023-06145-x 37587295

[ref15] Carstens N. Adejube B. Strunskus T. Faupel F. Brown S. Vahl A. (2022). Brain-like critical dynamics and long-range temporal correlations in percolating networks of silver nanoparticles and functionality preservation after integration of insulating matrix. Nanoscale Adv. 4, 3149–3160. doi: 10.1039/D2NA00121G, PMID: 36132822 PMC9418118

[ref16] Carving Out Brain Structure with Connectomics (2022). Biodock. Available at: https://blog.biodock.ai/electron-microscopy-and-connectomics/ (Accessed October 8, 2024).

[ref17] Chen X. Zhang J. Lin B. Chen Z. Wolter K. Min G. (2022). Energy-efficient offloading for DNN-based smart IoT Systems in Cloud-Edge Environments. IEEE Trans Parallel Distrib Syst 33, 683–697. doi: 10.1109/TPDS.2021.3100298

[ref18] Cybenko G. (1989). Approximation by superpositions of a sigmoidal function. Math. Control Signal Syst. 2, 303–314. doi: 10.1007/BF02551274

[ref19] Daniels R. K. Brown S. A. (2021). Nanowire networks: how does small-world character evolve with dimensionality? Nanoscale Horiz. 6, 482–488. doi: 10.1039/D0NH00693A, PMID: 33982039

[ref20] Daniels R. K. Mallinson J. B. Heywood Z. E. Bones P. J. Arnold M. D. Brown S. A. (2022). Reservoir computing with 3D nanowire networks. Neural Netw. 154, 122–130. doi: 10.1016/j.neunet.2022.07.001, PMID: 35882080

[ref21] De Vries A. (2023). The growing energy footprint of artificial intelligence. Joule 7, 2191–2194. doi: 10.1016/j.joule.2023.09.004

[ref22] Dosovitskiy A. Beyer L. Kolesnikov A. Weissenborn D. Zhai X. Unterthiner T.. (2021). An image is worth 16x16 words: transformers for image recognition at scale. Available at: http://arxiv.org/abs/2010.11929 (Accessed October 18, 2024).

[ref23] Dresp-Langley B. (2020). Seven properties of self-Organization in the Human Brain. BDCC 4:10. doi: 10.3390/bdcc4020010

[ref24] Elahi A. (2022). “Boolean logics and logic gates” in Computer systems: Digital design, fundamentals of computer architecture and ARM assembly language (Cham: Springer International Publishing), 33–50.

[ref25] Finocchio G. Incorvia J. A. C. Friedman J. S. Yang Q. Giordano A. Grollier J.. (2024). Roadmap for unconventional computing with nanotechnology. Nano Futures 8:012001. doi: 10.1088/2399-1984/ad299a

[ref26] Fostner S. Brown S. A. (2015). Neuromorphic behavior in percolating nanoparticle films. Phys. Rev. E 92:052134. doi: 10.1103/PhysRevE.92.052134, PMID: 26651673

[ref27] Gidon A. Zolnik T. A. Fidzinski P. Bolduan F. Papoutsi A. Poirazi P.. (2020). Dendritic action potentials and computation in human layer 2/3 cortical neurons. Science 367, 83–87. doi: 10.1126/science.aax6239, PMID: 31896716

[ref28] Haensch W. Raghunathan A. Roy K. Chakrabarti B. Phatak C. M. Wang C.. (2023). Compute in-memory with non-volatile elements for neural networks: a review from a co-design perspective. Adv. Mater. 35:e2204944. doi: 10.1002/adma.202204944, PMID: 36579797

[ref29] Harris J. J. Jolivet R. Attwell D. (2012). Synaptic energy use and supply. Neuron 75, 762–777. doi: 10.1016/j.neuron.2012.08.019 22958818

[ref30] Häusser M. Spruston N. Stuart G. J. (2000). Diversity and dynamics of dendritic signaling. Science 290, 739–744. doi: 10.1126/science.290.5492.739 11052929

[ref31] Herculano-Houzel S. (2009). The human brain in numbers: a linearly scaled-up primate brain. Front. Hum. Neurosci. 3:31. doi: 10.3389/neuro.09.031.2009, PMID: 19915731 PMC2776484

[ref32] Hopfield J. J. (1982). Neural networks and physical systems with emergent collective computational abilities. Proc. Natl. Acad. Sci. USA 79, 2554–2558. doi: 10.1073/pnas.79.8.2554, PMID: 6953413 PMC346238

[ref33] Huang H.-M. Wang Z. Wang T. Xiao Y. Guo X. (2020). Artificial neural networks based on Memristive devices: from device to system. Adv. Intell. Syst. 2:2000149. doi: 10.1002/aisy.202000149

[ref34] Ielmini D. Ambrogio S. (2020). Emerging neuromorphic devices. Nanotechnology 31:092001. doi: 10.1088/1361-6528/ab554b 31698347

[ref35] Indiveri G. Linares-Barranco B. Hamilton T. J. van Schaik A. Etienne-Cummings R. Delbruck T.. (2011). Neuromorphic silicon neuron circuits. Front. Neurosci. 5:73. doi: 10.3389/fnins.2011.00073, PMID: 21747754 PMC3130465

[ref36] Indiveri G. Linares-Barranco B. Legenstein R. Deligeorgis G. Prodromakis T. (2013). Integration of nanoscale memristor synapses in neuromorphic computing architectures. Nanotechnology 24:384010. doi: 10.1088/0957-4484/24/38/384010, PMID: 23999381

[ref37] Jaeger H. (2021). Towards a generalized theory comprising digital, neuromorphic and unconventional computing. Neuromorph. Comput. Eng. 1:012002. doi: 10.1088/2634-4386/abf151

[ref38] Jaeger H. Noheda B. Van Der Wiel W. G. (2023). Toward a formal theory for computing machines made out of whatever physics offers. Nat. Commun. 14:4911. doi: 10.1038/s41467-023-40533-1, PMID: 37587135 PMC10432384

[ref39] Kelso J. A. S. (2012). Multistability and metastability: understanding dynamic coordination in the brain. Philos. Trans. R. Soc. B 367, 906–918. doi: 10.1098/rstb.2011.0351, PMID: 22371613 PMC3282307

[ref40] Kia B. Lindner J. F. Ditto W. L. (2017). Nonlinear dynamics as an engine of computation. Philos. Trans. R. Soc. A Math. Phys. Eng. Sci. 375:20160222. doi: 10.1098/rsta.2016.0222, PMID: 28115619 PMC5311441

[ref41] Kitano H. (2004). Biological robustness. Nat. Rev. Genet. 5, 826–837. doi: 10.1038/nrg1471 15520792

[ref42] Kozachkov L. Lundqvist M. Slotine J.-J. Miller E. K. (2020). Achieving stable dynamics in neural circuits. PLoS Comput. Biol. 16:e1007659. doi: 10.1371/journal.pcbi.1007659, PMID: 32764745 PMC7446801

[ref43] Kozma R. Ilin R. Siegelmann H. T. (2018). Evolution of abstraction across layers in deep learning neural networks. Proc. Comput. Sci. 144, 203–213. doi: 10.1016/j.procs.2018.10.520

[ref44] Kuncic Z. Nakayama T. (2021). Neuromorphic nanowire networks: principles, progress and future prospects for neuro-inspired information processing. Adv. Phys. X 6:1894234. doi: 10.1080/23746149.2021.1894234

[ref45] Larkum M. E. Zhu J. J. Sakmann B. (1999). A new cellular mechanism for coupling inputs arriving at different cortical layers. Nature 398, 338–341. doi: 10.1038/18686, PMID: 10192334

[ref46] LeCun Y. Bengio Y. Hinton G. (2015). Deep learning. Nature 521, 436–444. doi: 10.1038/nature14539 26017442

[ref47] Legenstein R. Maass W. (2011). Branch-specific plasticity enables self-Organization of Nonlinear Computation in single neurons. J. Neurosci. 31, 10787–10802. doi: 10.1523/JNEUROSCI.5684-10.2011, PMID: 21795531 PMC6623094

[ref48] Li H. Wang S. Zhang X. Wang W. Yang R. Sun Z.. (2021). Memristive crossbar arrays for storage and computing applications. Adv. Intell. Syst. 3:2100017. doi: 10.1002/aisy.202100017

[ref49] Loomba S. Straehle J. Gangadharan V. Heike N. Khalifa A. Motta A.. (2022). Connectomic comparison of mouse and human cortex. Science 377:eabo0924. doi: 10.1126/science.abo0924, PMID: 35737810

[ref50] Luscher C. Malenka R. C. (2012). NMDA receptor-dependent long-term potentiation and long-term depression (LTP/LTD). Cold Spring Harb. Perspect. Biol. 4:a005710. doi: 10.1101/cshperspect.a005710, PMID: 22510460 PMC3367554

[ref51] Mäki-Marttunen T. Mäki-Marttunen V. (2022). Excitatory and inhibitory effects of HCN channel modulation on excitability of layer V pyramidal cells. PLoS Comput. Biol. 18:e1010506. doi: 10.1371/journal.pcbi.1010506, PMID: 36099307 PMC9506642

[ref52] Mallinson J. B. Shirai S. Acharya S. K. Bose S. K. Galli E. Brown S. A. (2019). Avalanches and criticality in self-organized nanoscale networks. Sci. Adv. 5:eaaw8438. doi: 10.1126/sciadv.aaw8438, PMID: 31700999 PMC6824861

[ref53] Marelli M. Divitini G. Collini C. Ravagnan L. Corbelli G. Ghisleri C.. (2011). Flexible and biocompatible microelectrode arrays fabricated by supersonic cluster beam deposition on SU-8. J. Micromech. Microeng. 21:045013. doi: 10.1088/0960-1317/21/4/045013

[ref54] Martini G. Reprogrammable threshold logic gates based on random nanostructured networks for algebraic and logic Boolean computation: Università degli Studi di Milano (Unpublished).

[ref55] Martini G. Mirigliano M. Paroli B. Milani P. (2022). The Receptron: a device for the implementation of information processing systems based on complex nanostructured systems. Jpn. J. Appl. Phys. 61:SM0801. doi: 10.35848/1347-4065/ac665c

[ref56] Martini G. Tentori E. Mirigliano M. Galli D. E. Milani P. Mambretti F. (2024). Efficiency and controllability of stochastic boolean function generation by a random network of non-linear nanoparticle junctions. Front. Phys. 12:1400919. doi: 10.3389/fphy.2024.1400919

[ref57] Masanet E. Shehabi A. Lei N. Smith S. Koomey J. (2020). Recalibrating global data center energy-use estimates. Science 367, 984–986. doi: 10.1126/science.aba3758, PMID: 32108103

[ref58] McCulloch W. S. Pitts W. (1943). A logical calculus of the ideas immanent in nervous activity. Bull. Math. Biophys. 5, 115–133. doi: 10.1007/BF02478259 2185863

[ref59] Mead C. (1990). Neuromorphic electronic systems. Proc. IEEE 78, 1629–1636. doi: 10.1109/5.58356

[ref60] Migliore M. Shepherd G. M. (2002). Emerging rules for the distributions of active dendritic conductances. Nat. Rev. Neurosci. 3, 362–370. doi: 10.1038/nrn810, PMID: 11988775

[ref61] Migliorini L. Piazzoni C. Pohako-Esko K. Di Girolamo M. Vitaloni A. Borghi F.. (2021). All-printed green Micro-supercapacitors based on a natural-derived ionic liquid for flexible transient electronics. Adv. Funct. Mater. 31:2102180. doi: 10.1002/adfm.202102180

[ref62] Migliorini L. Santaniello T. Borghi F. Saettone P. Comes Franchini M. Generali G.. (2020). Eco-friendly supercapacitors based on biodegradable poly(3-Hydroxy-butyrate) and ionic liquids. Nano 10:2062. doi: 10.3390/nano10102062, PMID: 33086532 PMC7603249

[ref63] Migliorini L. Santaniello T. Falqui A. Milani P. (2023). Super-stretchable resistive strain sensor based on Ecoflex–gold nanocomposites. ACS Appl. Nano Mater. 6, 8999–9007. doi: 10.1021/acsanm.3c01614

[ref64] Milano G. Cultrera A. Boarino L. Callegaro L. Ricciardi C. (2023). Tomography of memory engrams in self-organizing nanowire connectomes. Nat. Commun. 14:5723. doi: 10.1038/s41467-023-40939-x, PMID: 37758693 PMC10533552

[ref65] Milano G. Miranda E. Ricciardi C. (2022a). Connectome of memristive nanowire networks through graph theory. Neural Netw. 150, 137–148. doi: 10.1016/j.neunet.2022.02.022, PMID: 35313246

[ref66] Milano G. Pedretti G. Fretto M. Boarino L. Benfenati F. Ielmini D.. (2020). Brain-inspired structural plasticity through reweighting and rewiring in multi-terminal self-organizing Memristive nanowire networks. Adv. Intell. Syst. 2:2000096. doi: 10.1002/aisy.202000096

[ref67] Milano G. Pedretti G. Montano K. Ricci S. Hashemkhani S. Boarino L.. (2022b). In materia reservoir computing with a fully memristive architecture based on self-organizing nanowire networks. Nat. Mater. 21, 195–202. doi: 10.1038/s41563-021-01099-9, PMID: 34608285

[ref68] Miller J. F. (2019). The alchemy of computation: designing with the unknown. Nat. Comput. 18, 515–526. doi: 10.1007/s11047-019-09738-6

[ref69] Minsky M. Papert S. A. (2017). Perceptrons: an introduction to computational geometry. Cambridge, Massachusetts: The MIT Press.

[ref70] Mirigliano M. Borghi F. Podestà A. Antidormi A. Colombo L. Milani P. (2019). Non-ohmic behavior and resistive switching of au cluster-assembled films beyond the percolation threshold. Nanoscale Adv. 1, 3119–3130. doi: 10.1039/C9NA00256A, PMID: 36133584 PMC9417734

[ref71] Mirigliano M. Decastri D. Pullia A. Dellasega D. Casu A. Falqui A.. (2020). Complex electrical spiking activity in resistive switching nanostructured au two-terminal devices. Nanotechnology 31:234001. doi: 10.1088/1361-6528/ab76ec, PMID: 32202254

[ref72] Mirigliano M. Milani P. (2021). Electrical conduction in nanogranular cluster-assembled metallic films. Adv. Phys. X 6:1908847. doi: 10.1080/23746149.2021.1908847

[ref73] Mirigliano M. Paroli B. Martini G. Fedrizzi M. Falqui A. Casu A.. (2021). A binary classifier based on a reconfigurable dense network of metallic nanojunctions. Neuromorph. Comput. Eng. 1:024007. doi: 10.1088/2634-4386/ac29c9

[ref74] Moldwin T. Segev I. (2020). Perceptron learning and classification in a modeled cortical pyramidal cell. Front. Comput. Neurosci. 14:33. doi: 10.3389/fncom.2020.00033, PMID: 32390819 PMC7193948

[ref75] Nadalini G. Borghi F. Košutová T. Falqui A. Ludwig N. Milani P. (2023). Engineering the structural and electrical interplay of nanostructured au resistive switching networks by controlling the forming process. Sci. Rep. 13:19713. doi: 10.1038/s41598-023-46990-4, PMID: 37953278 PMC10641076

[ref76] Nakajima K. (2020). Physical reservoir computing—an introductory perspective. Jpn. J. Appl. Phys. 59:060501. doi: 10.35848/1347-4065/ab8d4f

[ref77] Neumann J. V. (1956). “Probabilistic logics and the synthesis of reliable organisms from unreliable components” in Automata Studies. (AM-34). eds. Shannon C. E. McCarthy J. (Princeton, New Jersey: Princeton University Press), 43–98.

[ref78] Nieus T. Sola E. Mapelli J. Saftenku E. Rossi P. D’Angelo E. (2006). LTP regulates burst initiation and frequency at mossy Fiber–granule cell synapses of rat cerebellum: experimental observations and theoretical predictions. J. Neurophysiol. 95, 686–699. doi: 10.1152/jn.00696.2005, PMID: 16207782

[ref79] Olivieri A. C. (2024). “Non-linearity and artificial neural networks. Multi-layer perceptron” in Introduction to multivariate calibration: A practical approach (Cham: Springer International Publishing), 271–288. doi: 10.1007/978-3-031-64144-2_14

[ref80] Padamsey Z. Rochefort N. L. (2023). Paying the brain’s energy bill. Curr. Opin. Neurobiol. 78:102668. doi: 10.1016/j.conb.2022.102668, PMID: 36571958

[ref81] Paroli B. Martini G. Potenza M. A. C. Siano M. Mirigliano M. Milani P. (2023). Solving classification tasks by a receptron based on nonlinear optical speckle fields. Neural Netw. 166, 634–644. doi: 10.1016/j.neunet.2023.08.001, PMID: 37604074

[ref82] Paulsen O. (2000). Natural patterns of activity and long-term synaptic plasticity. Curr. Opin. Neurobiol. 10, 172–180. doi: 10.1016/S0959-4388(00)00076-3, PMID: 10753798 PMC2900254

[ref83] Pfaff D. W. Volkow N. D. Rubenstein J. L. (Eds.) (2022). Neuroscience in the 21st century: From basic to clinical. Cham: Springer International Publishing.

[ref84] Previdi A. Piazzoni C. Borghi F. Schulte C. Lorenzelli L. Giacomozzi F.. (2021). Micropatterning of substrates for the culture of cell networks by stencil-assisted additive nanofabrication. Micromachines 12:94. doi: 10.3390/mi12010094, PMID: 33477416 PMC7829752

[ref85] Profumo F. Borghi F. Falqui A. Milani P. (2023). Potentiation and depression behaviour in a two-terminal memristor based on nanostructured bilayer ZrOx/au films. J. Phys. D. Appl. Phys. 56:355301. doi: 10.1088/1361-6463/acd704

[ref86] Radice S. Profumo F. Borghi F. Falqui A. Milani P. (2024). Programmable analog circuits with neuromorphic nanostructured platinum films. Adv. Electron. Mater.:2400434. doi: 10.1002/aelm.202400434

[ref87] Richards B. A. Lillicrap T. P. (2022). The brain-computer metaphor debate is useless: a matter of semantics. Front. Comput. Sci. 4:810358. doi: 10.3389/fcomp.2022.810358

[ref88] Rosenblatt F. (1958). The perceptron: a probabilistic model for information storage and organization in the brain. Psychol. Rev. 65, 386–408. doi: 10.1037/h0042519, PMID: 13602029

[ref89] Rosenblatt F. (1962). Principles of Neurodynamics: Perceptrons and the theory of brain mechanisms. Washington, D.C: Spartan Books.

[ref90] Rumelhart D. E. Hinton G. E. Williams R. J. (1986). Learning representations by back-propagating errors. Nature 323, 533–536. doi: 10.1038/323533a0

[ref91] Schuman C. D. Kulkarni S. R. Parsa M. Mitchell J. P. Date P. Kay B. (2022). Opportunities for neuromorphic computing algorithms and applications. Nat. Comput. Sci. 2, 10–19. doi: 10.1038/s43588-021-00184-y, PMID: 38177712

[ref92] Shapson-Coe A. Januszewski M. Berger D. R. Pope A. Wu Y. Blakely T.. (2024). A petavoxel fragment of human cerebral cortex reconstructed at nanoscale resolution. Science 384:eadk4858. doi: 10.1126/science.adk4858, PMID: 38723085 PMC11718559

[ref93] Skarda C. A. Freeman W. J. (1987). How brains make chaos in order to make sense of the world. Behav. Brain Sci. 10, 161–173. doi: 10.1017/S0140525X00047336

[ref94] Sporns O. Tononi G. Kötter R. (2005). The human connectome: a structural description of the human brain. PLoS Comput. Biol. 1:e42. doi: 10.1371/journal.pcbi.0010042, PMID: 16201007 PMC1239902

[ref95] Spruston N. (2008). Pyramidal neurons: dendritic structure and synaptic integration. Nat. Rev. Neurosci. 9, 206–221. doi: 10.1038/nrn2286 18270515

[ref96] Stöckel A. Eliasmith C. (2021). Passive nonlinear dendritic interactions as a computational resource in spiking neural networks. Neural Comput. 33, 96–128. doi: 10.1162/neco_a_01338, PMID: 33080158

[ref97] Suárez L. E. Markello R. D. Betzel R. F. Misic B. (2020). Linking structure and function in macroscale brain networks. Trends Cogn. Sci. 24, 302–315. doi: 10.1016/j.tics.2020.01.008, PMID: 32160567

[ref98] Tafreshi H. V. Benedek G. Piseri P. Vinati S. Barborini E. Milani P. (2001). Aerodynamic focusing of clusters into a high intensity and low divergence supersonic beam. Eur. Phys. J. AP 16, 149–156. doi: 10.1051/epjap:2001204

[ref99] Tanaka G. Yamane T. Héroux J. B. Nakane R. Kanazawa N. Takeda S.. (2019). Recent advances in physical reservoir computing: a review. Neural Netw. 115, 100–123. doi: 10.1016/j.neunet.2019.03.005, PMID: 30981085

[ref100] Teuscher C. (2014). Unconventional computing catechism. Front. Robot. AI 1:10. doi: 10.3389/frobt.2014.00010

[ref101] Tian Y. Sun P. (2022). Percolation may explain efficiency, robustness, and economy of the brain. Netw. Neurosci. 6, 765–790. doi: 10.1162/netn_a_00246, PMID: 36605416 PMC9810365

[ref102] Tsodyks M. Wu S. (2013). Short-term synaptic plasticity. Scholarpedia 8:3153. doi: 10.4249/scholarpedia.3153

[ref103] Turing A. M. (1937). On computable numbers, with an application to the Entscheidungsproblem. Proc. Lond. Math. Soc. s2-42, 230–265. doi: 10.1112/plms/s2-42.1.230

[ref104] Usami Y. van de Ven B. Mathew D. G. Chen T. Kotooka T. Kawashima Y.. (2021). In-Materio reservoir computing in a sulfonated polyaniline network. Adv. Mater. 33:e2102688. doi: 10.1002/adma.202102688, PMID: 34533867 PMC11469268

[ref105] Vahl A. Milano G. Kuncic Z. Brown S. A. Milani P. (2024). Brain-inspired computing with self-assembled networks of nano-objects. J. Phys. D. Appl. Phys. 57:503001. doi: 10.1088/1361-6463/ad7a82

[ref106] Vaswani A. Shazeer N. Parmar N. Uszkoreit J. Jones L. Gomez A. N.. (2017). Attention is all you need. arXiv: 1706.03762v7. doi: 10.48550/ARXIV.1706.03762

[ref107] Vingron S. P. (2024). Logic circuit design: Selected topics and methods. Cham: Springer International Publishing.

[ref108] Xu-Friedman M. A. Regehr W. G. (2003). Ultrastructural contributions to desensitization at cerebellar mossy fiber to granule cell synapses. J. Neurosci. 23, 2182–2192. doi: 10.1523/JNEUROSCI.23-06-02182.2003, PMID: 12657677 PMC6742013

[ref109] Yan M. Huang C. Bienstman P. Tino P. Lin W. Sun J. (2024). Emerging opportunities and challenges for the future of reservoir computing. Nat. Commun. 15:2056. doi: 10.1038/s41467-024-45187-1, PMID: 38448438 PMC10917819

[ref110] Zhang R. Gupta P. Zhong L. Jha N. K. (2008). “Synthesis and optimization of threshold logic networks with application to nanotechnologies” in Design, automation, and test in Europe. eds. Lauwereins R. Madsen J. (Dordrecht: Springer Nature), 325–343.

